# Targeting the osteosarcoma cancer stem cell

**DOI:** 10.1186/1749-799X-5-78

**Published:** 2010-10-27

**Authors:** Valerie A Siclari, Ling Qin

**Affiliations:** 1 Department of Orthopaedic Surgery, University of Pennsylvania School of Medicine, Philadelphia, PA, USA

## Abstract

Osteosarcoma is the most common type of solid bone cancer and the second leading cause of cancer-related death in pediatric patients. Many patients are not cured by the current osteosarcoma therapy consisting of combination chemotherapy along with surgery and thus new treatments are urgently needed. In the last decade, cancer stem cells have been identified in many tumors such as leukemia, brain, breast, head and neck, colon, skin, pancreatic, and prostate cancers and these cells are proposed to play major roles in drug resistance, tumor recurrence, and metastasis. Recent studies have shown evidence that osteosarcoma also possesses cancer stem cells. This review summarizes the current knowledge about the osteosarcoma cancer stem cell including the methods used for its isolation, its properties, and its potential as a new target for osteosarcoma treatment.

## Introduction

Osteosarcoma is the most common type of solid bone cancer, mainly arising in children and young adults. About 6 in every million children and 2 in every million adults will develop osteosarcoma [1]. Osteosarcomas most commonly develop in the long bones, in particular the distal femur and proximal tibia. They are often very aggressive (high-grade tumors) with about 20% of patients presenting with metastases. Osteosarcomas most commonly metastasize to the lung but also can metastasize locally to other sites within the bone. Osteosarcomas are characterized as tumors that produce osteoid. By X-ray, osteosarcomas often appear as tumors associated with mixed osteolytic and osteoblastic bone destruction and a soft tissue mass. They can be histologically classified into three types: osteoblastic, chondroblastic, and fibroblastic (reviewed in [2,3]). Microarray analysis has revealed that there are significant gene expression differences amongst the sub-types. 172 genes were differentially expressed between osteoblastic and non-osteoblastic osteosarcomas [4].

Osteosarcoma is believed to arise from mesenchymal stem cells (MSCs) or osteoprogenitor cells due to a disruption in the osteoblast differentiation pathway [5,6]. Genetic instability has made identifying the cause(s) of osteosarcoma development difficult [7]. A number of pathways and inactivating mutations have been proposed to play a role in osteosarcoma development including downregulation of the Wnt signaling pathway and inactivating mutations in p53 and retinoblastoma. However, none of these pathways/mutations have been implicated as main causes of osteosarcoma [2,6,8]. Paget's disease and prior irradiation are also risk factors for osteosarcoma [9]. In a study comparing the gene expression of 22 human osteosarcoma tumors to 5 normal human osteoblasts, osteosarcoma tumors had increased expression of RECQL4, SPP1, RUNX2, and IBSP and decreased DOCK5, CDKN1A, RB1, P53, AND LSAMP compared to normal osteoblasts. Increased Runx2 expression was associated with a poor response to chemotherapy [10]. High expression of the cell cycle inhibitor p21/WAF1 has also been proposed to indicate a worse prognosis [11].

Since the 1970s, combination chemotherapy along with limb-sparing surgery has been the main treatment for osteosarcoma. The most commonly used chemotherapeutic regimen includes pre- and post-operative cisplatin and doxorubicin with or without high-dose methotrexate [3]. Many patients develop resistance to this current therapy and tumor recurrence. Five-year patient survival has plateaued at about 70% for patients with non-metastatic disease and outcome is much worse for patients with metastases [2,12]. Targeting molecules important for tumorigenesis, "targeted therapy", has been an exciting development in cancer treatment in the past ten years. Yet, no such therapy is currently available for osteosarcoma. Today, osteosarcoma remains the second leading cause of cancer-related death for children and young adults [13] and therefore, there is a great need for developing new osteosarcoma treatments.

### The Cancer Stem Cell Hypothesis

The cancer stem cell hypothesis proposes that within a heterogeneous tumor there is a small subpopulation of cells called "cancer stem cells (CSCs)" that are responsible for forming the bulk of the tumor [14-16]. They are similar to stem cells and may arise from the transformation of stem cells or the de-differentiation of non-stem cells. They are quiescent and capable of both self-renewal and differentiation into all of the cells within a tumor.

The first evidence of the existence of CSCs came from studies of hematological malignancies. In 1994, Lapidot and colleagues showed evidence that only a small percentage of acute myeloid leukemia (AML) cells were capable of initiating leukemia in mice [17]. They found that at least 250,000 peripheral blood cells from AML patients were required for leukemic engraftment in severe combined immunodeficiency (SCID) mice, suggesting that there was only 1 cell per 250,000 cells capable of engraftment. Using fluorescence-activated cell sorting (FACS), leukemic stem cells were isolated as a subpopulation of less than 0.2% of the total leukemic cells in AML patients with similar cell surface markers (CD34^+^CD38^-^) to normal hematopoietic stem cells [17,18]. Interestingly, only the CD34^+^CD38^- ^leukemic stem cell population but not the CD34^+^CD38^+ ^or CD34^- ^population was able to form AML in SCID mice.

Following the success in hematological malignancies, FACS and magnetic-activated cell sorting (MACS) for stem cell surface markers including CD34, CD138, CD20, CD90, CD133, and CD44 have now been widely employed to identify CSCs in a number of cancers (reviewed in [15,19]). However, the use of tissue-specific stem cell markers to identify CSCs is limited by the lack of knowledge of these markers for every tissue type. Other methods to isolate CSCs are based on common characteristics of normal stem cells. These include growth of cells in serum-free, non-adherent sphere assays, serial colony-forming unit assays, sorting of cells for aldehyde dehydrogenase (ALDH) activity, and sorting for side population (SP) cells [15,19]. Although these functional assays are great tools to determine if a population possesses stem cells when normal stem cell surface markers are unknown, one pitfall is that these assays mostly just enrich for CSCs and therefore actually provide a mixed population of cells for study. The best evidence that cells isolated through these methods are true cancer stem cells comes from serial transplantation studies in which sorted cells are grown in xenograft models (typically in non-obese diabetic/severe combined immunodefficiency (NOD/SCID) mice), resorted and retransplanted to form new tumors (reviewed in [14,15]).

Using the above mentioned assays, the presence of CSCs has now been identified not just in hematological malignancies but also in a number of solid tumors including breast, brain, skin, lung, colon, pancreatic, liver, head and neck, and prostate cancers [15]. Overall, the identified CSCs are a subpopulation (< 1%) of the overall tumor cell population [20] and have high tumorigenic potential, requiring much lower numbers of cells to form tumors in mice than non-CSCs (some showing as low as 100 cells being capable of forming tumors in mice) (reviewed in [14,15]). They not only regrow CSCs when transplanted into mice, but, reform the whole heterogeneous population of tumor cells within these xenograft models. They also have upregulation of genes associated with stem cell maintenance of self-renewal and pluripotency such as Oct4 and Nanog and drug transporters such as ABCG2 [21-26].

Similar to stem cells, evidence suggests that CSCs are resistant to cancer therapies including radiation and chemotherapy. For example, CD133^+ ^glioma stem cells are less sensitive to radiation and undergo less radiation-induced apoptosis than CD133^- ^glioma cells both in vitro and in vivo. In fact, radiation enriches the percentage of CD133^+ ^glioma stem cells relative to other tumor cells [27]. CD133^+ ^glioblastoma stem cells are more resistant to the chemotherapeutic agents temozolomide, carboplatin, paclitaxel and etoposide compared to CD133^- ^cells [28]. Neuroblastoma and mouse ovarian cancer SP cells are more resistant to chemotherapeutic agents than non-SP cells [29,30]. Paired breast cancer core biopsies obtained from patients with primary breast cancer before and after 12 weeks of chemotherapy found that chemotherapy caused a 3-fold increase in the CD44^+^/CD24^-/low ^breast CSC population [31]. CSC characteristics such as quiescence, increased drug-efflux ability, increased DNA repair ability, and increased resistance to apoptosis have been proposed to contribute to CSC resistance to cancer therapies [15]. Therefore, although treatment with chemotherapy or radiation may reduce the bulk of the tumor, it may actually miss the most important cell to target, the cancer stem cell. Following chemotherapy or radiation therapy, CSCs may survive and could begin to differentiate and reform the tumor. Hence, CSCs are proposed to be responsible for chemoresistance, tumor recurrence, and tumor progression in many tumor types [15,19].

Although CSCs may be resistant to chemotherapy, evidence from studies of leukemia has shown that it is possible to find drugs that specifically inhibit the growth of CSCs. For example, the anthracycline idarubicin in combination with the proteasome inhibitor MG-132 induced apoptosis of AML stem cells in vitro and in vivo with no effect on normal hematopoietic stem cell viability [32]. Another study found that parthenolide, an inhibitor of NFκb, had similar effects and inhibited tumorogenesis in mice [33].

Several methods have been proposed to target the CSC [15]. One method is targeting cytotoxic drugs to CSCs using stem cell surface markers. For example, targeting CD33 (an AML stem cell surface marker) with the FDA-approved drug gemtuzumab ozogamicin (Mylotarg), a recombinant humanized anti-CD33 monoclonal antibody conjugated to calicheamicin (a cytotoxic antibiotic), did produce some but low anti-leukemic activity in CD33^+ ^AML patients 60 years and older who are not eligible for other cytotoxic therapies [34]. Another method is to target the CSC microenvironment, such as the blood vessels in vascular niches. Treatment of U87 glioma cell xenografts with the anti-angiogenic inhibitor Bevacizumab (anti-vascular endothelial growth factor (VEGF) monoclonal antibody) significantly decreased the number of vessel-associated CD133^+^nestin^+ ^brain cancer stem cells in mice [35]. Induction of CSC differentiation could be another way to eliminate these cells. All-trans retinoic acid induced differentiation of leukemic cells and increased relapse-free and overall survival in acute promyelocytic leukemia patients when given prior to anthracycline treatment [36]. However, patients often quickly develop resistance to retinoids.

### Evidence for Cancer Stem Cells in Osteosarcoma

Since the proposal of the CSC hypothesis, many studies have been performed to identify the osteosarcoma CSC. Currently, there are three methods that have now been employed to enrich for osteosarcoma CSCs including: (1) the sphere culture assay (or sarcosphere assay), (2) cell sorting for CD133, high ALDH activity, SP cells, or CD117 in combination with Stro-1, and (3) identification of cells that express the embryonic stem cell gene Oct4. This review will summarize each of these methods below.

### 1. Sphere Culture Assay

Gibbs et al. (2005) were the first to show that osteosarcomas possess cells with CSC characteristics [37]. When grown in serum-free semi-solid N2 medium with epidermal growth factor (EGF) and fibroblast growth factor basic (FGFb) in low attachment plates, MG-63 human osteosarcoma cells and primary osteosarcoma cells formed spheres at a frequency of 0.1 to 1%. These spheres had increased expression of the embryonic stem cell markers Oct4 and Nanog compared to adherent cells. Osteosarcoma spheres also had self-renewal ability as dissociation of the spheres produced single cells capable of forming secondary spheres at an equal or higher rate than adherent cells. Consistent with these results, several other groups have also confirmed the ability of osteosarcomas to form spheres [38-40]. The human osteosarcoma cell lines OS99-1, Hu09, MG-63 and Saos-2 and the canine osteosarcoma cell lines D-17, UW0S-1, and UWOS-2 are all capable of forming spheres which express the embryonic stem cell genes Oct4 and Nanog and therefore have a primitive phenotype. In these experiments, spheres could be reproduced consistently when passaged multiple times and produced adherent cell cultures when returned to normal growth conditions. Interestingly, MG-63 spheres were less sensitive to doxorubicin and cisplatin than adherent cells and had increased expression of the DNA mismatch repair enzyme genes MLH1 and MSH2, suggesting that these sphere cells might confer chemoresistance [38,41].

### 2. Cell Sorting

#### A. CD133 (prominin-1)

CD133 (prominin-1) is a pentaspan membrane glycoprotein used initially as a marker for neuroepithelial stem cells and has been subsequently used as a marker for many CSCs including brain and colon CSCs [42-45]. Recently, Tirino et al. identified a small CD133^+ ^population (3-5%) in the human osteosarcoma cell lines MG-63, Saos-2, and U20S with stem cell characteristics [45]. Compared to CD133^- ^cells, these cells had an increased percentage of cells in G2/M phase, were Ki67-positive and had increased in vitro growth, indicating that they are more proliferative. CD133^+ ^cells, but not CD133^- ^cells, were capable of forming spheres in culture and had an increased ability to form colonies in a soft agar assay. Cells obtained from spheres formed by CD133^+ ^cells were capable of forming new spheres containing both CD133^- ^and CD133^+ ^cells, indicating that CD133^+ ^cells can differentiate into CD133^- ^cells. Spheres initially formed from CD133^+ ^cells and passaged 4 to 6 times showed increased expression of Oct4 and CD133. In addition to expressing CD133, the human osteosarcoma cell lines Saos-2, OSA-1, OSA-2, and OSA-3 also express nestin, a marker for neural stem cells and brain CSCs, suggesting that nestin and CD133 might be used as co-markers for identifying osteosarcoma CSCs [46].

#### B. Hoechst 33342 Dye Exclusion and the Side Population (SP) cells

SP cells are capable of effluxing the DNA-binding dye Hoechst 33342 using ATP-binding cassette (ABC) transporters. This ability to efflux Hoechst dye was first identified as a characteristic of normal haematopoietic stem cells [47,48] but has subsequently been used to identify CSCs in cancers such as gastrointestinal and ovarian cancer [30,49]. Murase and colleagues screened seven osteosarcoma cell lines including OS2000, KIKU, NY, Huo9, HOS, U20S, and Saos-2 cells for the presence of a side population [50]. Only the NY osteosarcoma cell line demonstrated a small percentage of cells (0.31%) with side population characteristics. However, the presence of stem cell characteristics in this population was not confirmed by the authors. Tirino et al. (2008) also attempted to identify SP cells in osteosarcoma. They found that CD133^+ ^Saos-2 cells do possess a small side population (0.97%) [45]. These results suggest that sorting for a side population alone is not a good technique to isolate the osteosarcoma CSC.

#### C. High Aldehyde Dehydrogenase (ALDH) Activity

ALDHs are a group of cytosolic enzymes that oxidize intracellular aldehydes into carboxylic acids [51]. High ALDH1 expression has been linked to leukemia, breast, and colon cancer chemoresistance [52-55]. Human and murine hematopoietic stem cells and neural stem and progenitor cells have increased ALDH activity compared to non-stem cells [56-58]. Detection of cells with high ALDH activity identifies CSCs in a number of cancers including breast, liver, colon, and acute myelogenous leukemia [59-62]. Wang et al. demonstrated that while adherent Hu09, Saos-2, and MG-63 cells possess small populations (1.8%, 1.6%, and 0.6% respectively) with high ALDH activity (ALDH(br)), OS99-1 contained a high percentage (45%) of ALDH(br) cells [63]. However, OS99-1 ALDH(br) cells isolated from cell cultures did not have increased tumorigenicity compared to cells with low ALDH activity (ALDH(lo)). Interestingly, growth in tumor xenografts dramatically decreased the ALDH(br) cell population in OS99-1 to less than 3%. These ALDH(br) cells from tumor xenografts had increased proliferation, colony formation ability, expression of the stem cell genes Oct4, Nanog, and Sox-2, and most importantly, increased tumorigenicity when subcutaneously injected into NOD/SCID mice compared to ALDH(lo) cells. Serial transplantation of these ALDH(br) cells showed that they were capable of self-renewal and reforming the bulk of the tumor. In contrast to the results of Wang et al., Honoki et al. showed a larger percentage of MG-63 cells (11%) with high ALDH activity. MG-63 sphere cells also were enriched for ALDH1 expression [41].

#### D. CD117 and Stro-1

CD117(c-kit) is the receptor for stem cell factor and a known proto-oncoprotein. It is also one of the markers used to isolate CSCs from ovarian cancer [64,65]. Stro-1 is a cell surface marker for mesenchymal stem cells [66]. Adhikari et al. found that sphere cells generated from the mouse osteosarcoma cell lines K7M2, 318-1, and P932 possessed characteristics of CSCs such as having increased tumorigenicity when injected subcutaneously into mice, increased expression of the drug transporter ABCG2, and an ability to differentiate into multiple lineages (osteogenic and adipogenic). The mouse sphere cells also had increased expression of the chemokine receptor CXCR4, a receptor linked to an increased metastatic ability, and an increased percentage of CD117^+^Stro-1^+ ^(DP) cells. DP K7M2 and 318-1 mouse osteosarcoma cells were more resistant to the chemotherapeutic doxorubicin than CD117^-^Stro-1^- ^(DN) and parental cells. Both mouse and human DP osteosarcoma cells had increased expression of ABCG2 and CXCR4 compared to DN cells. DP mouse 318-1, K7M2, and P932 and human KHOS, BCOS, and MNNG/HOS osteosarcoma cells had increased tumorigenicity when subcutaneously injected into nude mice compared to DN cells derived from the same cell line. 318-1 DP cells produced tumors not just with DP cells but also DN and single positive, suggesting that 318-1 DP cells not only self-renew but also can differentiate and reform all of the cells within the tumor. When 318-1 DP cells were injected into the femoral bone marrow cavity of NOD/SCID mice, they had increased primary tumor take and metastasis to the lung. These lung metastases had more cells positive for the markers CD117, Stro-1, ABCG2, and CXCR4 than the primary bone tumor [66], suggesting that the osteosarcoma CSCs are the cells with an increased ability to metastasize to lung.

### 3. Oct4

Oct4 is a central determinant of embryonic stem (ES) cell identity and one of four transcriptional factors which, when introduced together, were sufficient to reprogram differentiated fibroblasts to confer pluripotency indistinguishable from ES cells [67]. Based on the findings that osteosarcoma spheres had increased expression of Oct4, Levings et al. engineered an osteosarcoma cell line (OS521Oct-4p) that stably expressed a human Oct4 promoter-driven GFP reporter [68]. Twenty-four percent of the cells in culture and 67% of the cells in xenografted tumors were GFP positive. These Oct4/GFP^+ ^cells from xenograft tumors also expressed the MSC markers CD105 and ICAM-1. Moreover, GFP-enriched cells were more than 100 fold more tumorigenic than GFP-depleted cells, capable of forming subcutaneous tumors with less than 300 cells in NOD/SCID mice and metastasizing to lung. These cells could also differentiate and form Oct4/GFP^- ^cells.

Overall, the methods mentioned above show evidence that a subpopulation of osteosarcoma cells do exist with cancer stem cell characteristics. One interesting common feature of the CSCs derived from the different isolation methods is that they all have increased expression of genes required for ES cell maintenance (Oct4 and Nanog) [37,38,45,50,63]. This is consistent with previous findings that many types of CSCs, including ovarian, prostate, renal carcinoma and Ewing's sarcoma, highly express Oct4 and Nanog [21,22,24,26]. However, these genes are difficult to use as markers for isolation. Furthermore, most commonly available untransformed human osteosarcoma cell lines, such as Saos-2, MG-63, and U2OS cells, are difficult to grow in animal models, hindering further research to test the in vivo tumorigenic ability of isolated CSCs and confirm their stem cell nature [13].

### Cells of Origin for Osteosarcoma Cancer Stem Cells

CSCs have been proposed to arise either from the transformation of normal stem cells to cancerous stem cells or from the dedifferentiation and transformation of progenitor or terminally-differentiated cells to tumor cells with stem cell-like characteristics [14]. Osteosarcomas are proposed to be a "differentiation-flawed disease", resulting from genetic and epigenetic disruption of the osteoblast differentiation pathway [6]. Evidence for this includes that osteosarcoma cells are similar to the bone-forming cell, the osteoblast, since both of these cells produce osteoid, suggesting that osteosarcomas arise from osteoblasts or osteoprogenitors. Osteosarcomas also have histological variability, not only having osteoblastic regions but also chondroblastic or fibroblastic regions [69], indicating that the osteosarcoma cell of origin may be a cell with multipotent potential. Mesenchymal stem cells (MSCs) are multipotent stem cells found in adult bone marrow capable of differentiating into not only osteoblasts but also cartilage, fat, tendon, muscle, and marrow stroma and therefore tumors arising from MSCs could resemble the varied histology of osteosarcomas [70]. Bone marrow-derived MSCs can spontaneously undergo malignant transformation after long-term culture and result in fibrosarcoma formation in vivo [71]. Firefly-luciferase and Dsred-labeled adult mouse MSCs (a cell line derived after non-tumorigenic genetic manipulation and long-term culture of MSCs) formed osteosarcoma-like tumors in mice [72]. Loss of the Cdkn2 locus, aneuploidization, and translocations in MSCs are involved in their malignant transformation [5]. Complete loss of one of the proteins encoded in the cdkn2 locus, CDKN2A/p16, was associated with lower survival in 88 osteosarcoma patients [5]. Therefore, osteosarcomas may arise from either MSCs or osteoprogenitors.

Taking into account the CSC hypothesis, we propose that MSCs might be the cells of origin for osteosarcoma CSCs. Therefore, further understanding of the MSC may aid in the understanding of the osteosarcoma CSC. Currently the markers for isolating MSCs are controversial and not as defined as the hematopoietic stem cell (reviewed in [73]). One of the criteria that the International Society for Cellular Therapy proposed to define a MSC population is that the cells must be "greater than or equal to 95% positive for CD73 (ecto-5'-nucleotidase), CD90 (Thy-1), and CD105 (endoglin) with no more than 2% of the cells positive for CD34, CD45, CD11 b or CD14, CD19 or CD79alpha, and HLA-DR" (markers of hematopoietic progenitors, endothelial cells, monocytes, macrophages, B cell markers, and stimulated mesenchymal stem cells) [73]. Other proposed MSC markers include: CD44, CD49a, STRO-1, CD200, CD271, and CD146 [73]. Gibbs et al. found that the MSC markers Stro-1, CD105, and CD44 were expressed in 2-10%, 30-50%, and 75-100% of osteosarcoma cells in culture, respectively [37]. Tirino et al. (2008) showed that nearly 100% of MG-63, U20S and Saos-2 cells express the MSC markers CD90, CD44, and CD29 [45,74]. Only one of these proposed mesenchymal stem cell markers, Stro-1, has been used to successfully isolate osteosarcoma cells with CSC characteristics. Stro-1 in combination with CD117 isolated cells with CSC characteristics from mouse and human osteosarcoma cells [66]. However, since the majority of osteosarcoma cells are positive for many of these proposed MSC markers, markers such as CD90, CD44, and CD29 may not be useful markers to isolate the osteosarcoma CSC. Identifying the novel and specific markers for MSCs will aid in identifying the osteosarcoma CSC.

### Possible Niche for Osteosarcoma Cancer Stem Cells

Normal stem cells are found within niches (microenvironments) that support the stem cell. Stem cells and niche cells interact with each other via adhesion molecules and molecular signals that are important for maintenance of stem cell self-renewal, differentiation, and quiescence [75]. For example, hematopoietic stem cells depend on interactions with osteoblasts in osteoblastic niches and interactions with endothelial cells in vascular niches in the bone marrow to maintain their stem cell characteristics [20].

Like normal stem cells, CSCs also require a microenvironmental niche to maintain stemness. CSCs may form their own niche or take over normal stem cell niches [20,76,77]. There is evidence that brain tumor cells reside in vascular niches. The putative nestin^+^CD133^+ ^brain CSCs were found next to capillaries in brain tumors and adhere to endothelial cells [35]. Co-injection of CD133^+ ^human medulloblastoma cells with endothelial cells into mice increased tumor formation [35]. If CSCs require environmental signals and cell interactions within niches to maintain their stem cell properties, this suggests that when studying the cancer stem cell, the environment in which the cells are studied is very important. Differences in behavior of osteosarcoma CSCs grown in vitro compared to in vivo have been observed. For example, although in vivo the CSC is characterized by being quiescent, in vitro osteosarcoma CSCs are more proliferative than the non-CSCs [20,37]. OS99-1 cells isolated with high ALDH activity only had the behavior of CSCs when cells were isolated from subcutaneous tumors and not from adherent in vitro cultures [63]. Therefore, when studying CSCs, it may be important to only use models that as closely as possible recapitulate the normal environment.

The osteosarcoma CSC niche has not been defined. However, if osteosarcoma CSCs arise from MSCs, it is feasible that they may reside within the proposed MSC niche, a perivascular niche (reviewed in [73]). The location of MSCs within perivascular niches is proposed to support the migration of MSCs in response to injury or disease [73]. Similarly, location within a perivascular niche may support the metastasis of osteosarcomas to lung.

Since the local environment affects the behavior of CSCs, studying osteosarcoma CSCs in the context of its local environment, the bone, may be important for determining how to target osteosarcoma CSCs for treatment. The bone is a unique environment with properties that could alter the behavior of a CSC. For example, the bone is a hypoxic environment [78]. Activation of the hypoxia signaling pathway activates many pathways important for stem cell maintenance and, interestingly, hypoxia increases the number of brain CSCs [79]. Therefore, hypoxia might play a role in regulating osteosarcoma CSCs. The bone matrix is also rich in growth factors [80]. Alterations in bone remodeling due to the development of osteosarcoma could cause release of growth factors, such as transforming growth factor beta (TGFβ) or bone morphogenetic proteins (BMPs) that are capable of influencing stem cell maintenance. The TGFβ signaling pathway is upregulated in breast CSCs and its inhibition induced breast CSC differentiation in vitro [81]. BMPs induce differentiation of brain tumor stem cells in vivo [82]. BMPs may not have a similar effect on osteosarcomas since BMPS do not induce differentiation of osteosarcomas but promote growth in vivo [83]. Bone also contains the chemokine ligand SDF-1 [84] and osteosarcomas express its receptor, CXCR4 [85]. The CXCR4/SDF-1 signaling pathway is involved in the maintenance of hematopoietic stem cell numbers [86]. Interaction of bone matrix-derived SDF-1 with CXCR4 receptors could be involved in maintaining the osteosarcoma CSC.

The orthotopic osteosarcoma model is produced by injecting osteosarcoma cells into the long bones of immuno-compromised mice. Despite the importance of the local environment in CSC behavior, to date, only one group has published results looking at the growth of potential osteosarcoma CSCs in an orthotopic model [66]. Adhikara et al. showed the difference in growth of CD117^+^Stro-1^+ ^mouse osteosarcoma cells compared to CD117^-^Stro-1^- ^cells in the femur of NOD/SCID mice. However, no one has studied human osteosarcoma CSCs in an orthotopic model. This is most likely because there are currently very few reports of untransformed human osteosarcoma cell lines that are commercially available and able to grow within this model [13]. Further development of either orthotopic osteosarcoma models or spontaneous osteosarcoma models is important for the study of the osteosarcoma CSC and its niche.

## Conclusions and Perspectives

**Figure 1:**
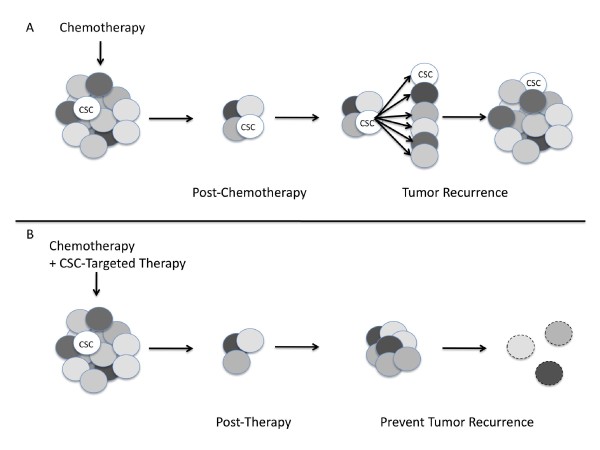
**The impact of the osteosarcoma cancer stem cell model on future treatment design**. (A) The response of osteosarcoma to chemotherapy alone: Chemotherapy shrinks the bulk of the tumor. However, chemoresistant CSCs may survive this therapy and then can self-renew and differentiate to reform the bulk of the tumor. CSCs therefore are responsible for osteosarcoma chemoresistance and tumor recurrence. (B) The proposed response of osteosarcoma to a combination of chemotherapy and CSC-targeted therapy: Combinational treatment will not only kill the majority of tumor cells but also the CSCs. The remaining non-CSC tumor cells will eventually exhaust their growth ability, resulting in complete eradication of the tumor.

There is compelling evidence that osteosarcoma tumors possess cancer stem cells. This will have a great impact on the design and evaluation of novel treatments for osteosarcoma. The current treatment, chemotherapy together with surgical removal, can only cure around 70% of osteosarcoma patients because of chemoresistance [2,12]. Osteosarcoma CSCs are proposed to be responsible for this chemoresistance and therefore should be considered as a major target for developing novel treatments (Figure 1) [2,12,38,87]. Current treatment with chemotherapy shrinks the bulk of the tumor but osteosarcoma CSCs remain unharmed. Following treatment, these CSCs can self-renew and reform the bulk of the tumor leading to tumor recurrence (Figure 1A). However, if a CSC-targeted therapy is incorporated, CSCs would be killed, eliminating the cells capable of reforming the bulk of the tumor. Post-therapy, any remaining non-CSCs could divide, but unlike CSCs, non-CSCs have limited proliferative capacity and would eventually die out (Figure 1B). Moreover, since preliminary animal data suggest that there are more CSCs in lung metastasis samples and that CSCs have an increased ability to metastasize to the lung [66], CSC-targeted therapy could also be an effective treatment to reduce osteosarcoma lung metastases. Therefore, we propose that a combination of chemotherapy, CSC-targeted therapy, and surgical removal of tumor will improve patient outcomes.

In order to develop CSC-targeted therapy, it is important to be able to specifically isolate the CSCs. Although the methods utilized to detect the osteosarcoma CSC show populations with enriched stem cell-like characteristics, no specific markers for the osteosarcoma CSC have been established. One immediate question is: What are the correct markers to isolate the osteosarcoma CSC? Further understanding of the MSC, a putative cell-of-origin for the osteosarcoma CSC, could aid in successful specific isolation of the osteosarcoma CSC.

Once we specifically isolate the osteosarcoma CSC, another question is: How can these cells be targeted and killed? One way to detect therapeutic targets in CSCs is to determine how these cells differ genetically from other non-CSCs using microarray analyses. One recent study found that MG-63 spheres have increased expression of the DNA repair enzyme genes MLH1 and MSH2 compared to adherent cells and increased resistance to the common osteosarcoma therapeutics cisplatin and doxorubicin [38]. Treatment of these spheres with caffeine, a DNA repair enzyme inhibitor, along with doxorubicin or cisplatin increased the inhibition of cell growth more than treatment with these chemotherapeutics alone. Therefore, the addition of drugs that increase sensitivity of the CSCs to current chemotherapy regimens could be important for the improvement of current therapy.

Although the CSC may be a great new target for cancer therapy, one major problem with the CSC as a therapeutic target is that it has many similar properties to normal stem cells. This leads to the third question: How do osteosarcoma CSCs differ from normal stem cells? It will be important to monitor the effect of proposed CSC therapeutics on normal stem cells to ensure a limited amount of non-specific toxicity. Further understanding of the osteosarcoma CSC will aid in determining how to target it. Microarray analyses can determine genes that are upregulated in the osteosarcoma CSC compared to non-CSCs but not in the normal stem cell population. High-throughput screening could identify drugs that CSCs are sensitive to, while leaving the normal stem cells unharmed. Ultimately, the development of new therapies targeting the osteosarcoma CSC requires the monitoring of any effect on normal stem cells as a potential side-effect.

## Competing interests

The authors declare that they have no competing interests.

## Authors' contributions

VS and LQ both reviewed the literature and decided upon the content of this review. VS wrote the first draft and both VS and LQ edited the manuscript. All authors read and approved the final manuscript.
